# Address

**Published:** 1859-11

**Authors:** J. Adams Allen

**Affiliations:** Prof. of Principles and Practice of Medicine


### ADDRESS

### Introductory to the Seventeenth Annual Course of Lectures in Rush Medical College,

DELIVERED NOV. 1, 1859.

*By* J. Adams Allen, *Prof. of Principles and Practice of Medicine.*

*Gentlemen of the Class:*

With this day commences the Seventeenth Regular Annual Session of the Rush Medical College, and it becomes my pleasant duty, on behalf of my colleagues of the faculty, to bid you cordial welcome.

In a country like ours, where the rattle of the rail car and the click of the telegraph intermingle with the sounds of the axe and the rifle of the pioneer, sixteen years of healthful life and activity give claim to the prerogatives of mature youth, if not of venerable age. Rush Medical College having passed beyond the years of doubtful experiment, and counting with pride its scores and hundreds of alumni engaged in honorable and successful practice of the principles here inculcated throughout the interior valley of the continent, and even upon its western slope to the Pacific, may well exult in the prosperity which has thus far attended its existence, the success which has crowned its teachings, and the brilliant future to which it may warrantably aspire.

We have invited you to these halls, Gentlemen, with the map of the country before us, with the volume of medical history in view, and, as we believe, not untaught as to the requirements of a science which is every day becoming broader and deeper, of an art which is every day becoming more exact and practical.

The metropolis of the North-West, concentrating the business energies, enterprise, wealth and population of a vast region of country unsurpassed, perhaps unapproachable by any other, in all the elements of agricultural, mineral, forest, manufacturing and commercial resources, offered, and still offers, a field for medical observation, experience and communication of their results, both scientifically and practically, which few localities afford. Here the union of science with art, the lecture and the clinic, can be realised, and become prolific of invaluable results.

Medicine, Gentlemen, is a progressive science; it has not reached the region of ultimate facts, around which, as central suns, systems of belief and practice can revolve in perpetual uniformity, undisturbed by the proximity of erratic truths. It accepts all truths, even though they refuse orderly arrangement. It explains them as best may be, but never submits to be governed by its explanation. The true medical man controls his theories, and is not controlled by them. We strive to bring medicine to the level of the exact sciences by guiding our inquiries, our experiments, our reasoning by the same inductive methods which have brought to light so many ultimate facts, or general laws outside the domain of medicine.

Incident to this attempt, a vast number of general laws, indubitable and exact, have been developed in the various sciences collateral to medicine. These are like the mathematical propositions which one by one for ages were being established, until by and by they proved the rounds of a ladder reaching from earth to the far away spheres, upon which Newton and his fellows have ascended to the ultimate principles of astronomy, and have plucked from the firmamental orbs the secret of gravitation.

Day by day the cultivators of our science are aiding in this great work, and, although it may not occur in our day, yet by the united opinion of the greatest names known to our science, we are warranted in believing that we stand upon the threshold of discoveries which shall forever release medicine from the imputation of being in any sense, "a conjectural art," and bring it upon the level of the exact sciences. This is the great object now before the professional world, and to the attainment of it certain qualifications are indispensable.

1. A clear, accurate and general acquaintance with all the known facts of medicine, including its collateral sciences, together with all their discoverable bearings upon each other.

2. A sound mind educated *how* to think, and free from subserviency to dogmatical injunctions of *what* to think.

3. An acquaintance with the history of the operations and results of other men's thoughts upon the same subjects.

Gentlemen, if any of you, in commencing the study of medicine, has been in any degree induced to take it up from a feeling of indolence fostered by the popular idea that in this science discrepancies of opinion are almost the rule, and therefore it is of trivial importance what particular ideas you do collect, you must allow me to disabuse your minds at once of this fatal error, by calling your attention to certain great classes of facts, which are as fixed and indubitable as nature affords, and of which you must attain a knowledge, even though you aspire but to a humble or mediocre place in the profession.

Thus it is seen that the Anatomical Structure of man constitutes a science of itself, though only a fraction of the medical cyclopædia. The shape, size, position, relations, general and minute structure of each part; the occasional diversities within the limits of health, are topics where all creeds and sects of medical men, whether surgeons or physicians, find common, indisputable and indispensable study.

Again, the body is surrounded by various materials, some of which in life it receives into its interior, and into which after death it is decomposed. All substances influence it, and all forces may come in contact with it, and how can the resulting effects be explained, unless both substances and forces be in themselves understood? Hence Chemistry in all its range and minutiæ—the scientific history of all ponderable and imponderable agencies must be investigated and understood as essential and constituent parts of the one great science of medicine.

But as in anatomy, the organs are to be studied, in repose, so it becomes necessary to study them in action. The prominent motions evincing animal life; the movements of fluids in their channels, as common or peculiar to animals and vegetables; the motions, transformations and conduct of the minute cells which the microscope brings into view; the changes which food and air undergo on being received within the body; the nature and source of animal heat; the nature and relations of the diverse fluids, their operations within and without; the development of the embryo to the adult, of cell and membrane and pulp to gland and nerve and muscle, of cartilage to bone; the separation of conjoint actions, and assignment of each to special organs; the mysterious organization through which the mental part develops its influence, and the modes of its manifestation; the influence of vital stimuli and all forms of force in promoting, retarding or modifying the action or composition of each or all of the organs; the determination of all influences, whether of form, connection, integral constitution, or of force, that maintain the equable and harmonious action of the several parts, and of the system entire. Surely here is the science for a life's pursuit.

But, as one has well said, "Had there been no other animals, the nature of man would have been far more difficult to be understood;" thus it is seen that philosophic Zoology is also to be studied. The internal and active organs of man are removed from the sphere of direct inquiries, save in rare and accidental cases. Hence we are led to search out the modes of action in animals having corresponding organs; or, if general likeness, but particular differences obtain, then to seek for the differences, and assign, if possible, the modified structure or conditions giving origin to them. Thus, little by little, we are led along, connecting structures and functions of the first simplicity, with structures and functions of the last complexity; contemplating life and organism, not in isolated instances, but in the linked series which they form, filling up one grand system, and rounding out the circle of animal life. And when, here and there, at intervals, a few links are found wanting in the grand chain, the very absence indicates the route of discovery to other instances, perhaps in the unexplored regions of the earth, or buried with its fossil forms, or yet to appear, of individuals that would complete the wonderful catalogue, and in the symmetrical plan of organization, fill up the vacant places.

Perhaps it is safe to say, that there is no other single science which at the present time is more minutely known, and in which the permanent trophies of inquiry are of more triumphant nature than in Physiology, or the science of life.

To the study of anatomy, chemistry and physiology, however, is yet to be joined that of disease or Pathology—that science which includes all we may know or apprehend of those changes in the structure or action, of parts or the whole, of the animal framework that impair perfection, or tend to dissolution. All of these, we believe, to be reducible to series and their causes to be developed. At one time variations of structure by direct injury, decomposing agents or poisons are seen to prevent the toward impression of vital stimuli; or these latter begin the difficulty, by similarly impairing structure. Now, the influence of general causes deranges the parts, and again deranged parts disturb the nice adjustment of the whole, and utter disorder ensues. Sometimes impaired action is noticed, with no manifest change of structure, or apparent modification of the sustaining forces of life. By a little examination it is seen that these apparently irregularly disordered actions and changes frequently fall into groups capable of being distinguished as peculiar, thus forming natural families with subordinate divisions. Each group attended, in the main, by special changes in the material constituents of the body, and preceded or accompanied by particular conditions, becomes as definite an object of study as newly discovered species of plants or animals, or new elements or compounds developed by the manipulations of the chemist.

Here and there a group may baffle even expert pathologists, and become a *questio vexata;* just as botanists, zoologists or chemists may have difficulty in assigning a habitation and name to some refractory members of their usually orderly families.

All questions here are upon the facts, to be settled as in all studies of nature by reference to the senses and their report. The subject matter is not within the appropriate range of hypothesis or theory; the laws of strict induction are here indispensable.

Theory may include all known facts, and hypothesis may apparently account for them, but to establish a law it becomes necessary to prove that the relation of the facts is not only general but *essential*. Theory can govern no farther than the instances actually examined and included, whereas a law invariably includes all the vast number of unexamined instances.

Thus, of the great number of theories invented since time immemorial to include the causes of disease, and the changes involved, it is sufficient to say that they were the product of a knowledge of facts merely without instances contradictory, which may be almost innumerable, and yet the systems based upon them be liable to overthrow at any moment by the discovery of new facts. And indeed this may be freely admitted to be the case with all preceding and all present theories upon this subject; nevertheless the discovered facts remain unchanged, and many of them have been referred to laws that can no more be shaken than any other demonstrated laws of nature.

The signs by which disease is discovered are daily becoming more accurately defined; fanciful distinctions are being discarded, and real ones pointed out; causes are being investigated and made known; changes in structure and action are progressively detected with greater minuteness; and thus pathology advances with steady step to the goal of scientific perfection. To this general statement, it is no objection to urge that in many details there is a complexity which, thus far, has eluded analysis. The wonder is, not that so much remains mysterious, but that in such a confessedly obscure subject, so much has been wrested from the domain of ignorance and darkness. Consideration of what is really known upon this topic, notwithstanding the tortuous path by which discovery has wended its way, may well impress the sentiment:

> "Trust not the frantic or mysterious guide,
> Nor stoop a captive to the schoolmen's pride;
> On nature's wonders fix alone thy zeal,
> They dim not reason."

Acquaintance with the branches of medical science adverted to will infallibly suggest numerous particulars, observance of which will ward off or prevent the incursions of disease. Practically the whole art of Hygiene springs as naturally from them as a tree from its roots.

Thus far, Gentlemen, you will perceive that I have made no allusion to the use of artificial curative agencies, whether medicinal or surgical. Indeed, it is quite possible to perform the higher parts of a physician's duty without recurring to special medicines; those who suppose that the art of medicine consists solely, or even in greatest part in the administration of drugs, entertain but a limited conception of its real character. And as it is precisely here that the greatest discrepancies of opinion occur, you will readily perceive that you have enough to occupy your time and attention for a long period yet, before you will reach the debateable regions of the science.

*Oportet discentem credere—oportet edoctum judicare*—is a maxim emphatically true in medical science. To be able to form opinions of any value you must stand upon a level with, or above, the facts. This is the simple, common sense reason, why what we call the regular or scientific physician will not stoop to hold controversy with the manifold shapes of quackery and charlatanism, in which ignorance vainly disguises itself. Not from any unwillingness to acquire new truths, but because opinions or doctrines based upon knowledge of a few, or perhaps upon no facts, though they may happen to be true, are infinitely more likely to be false and absurd.

Many persons seem to think that it is a mark of weakness not to have a full fledged opinion, upon any subject that is brought to their notice. They will decide the profoundest questions in ethics, politics, law or medicine, with an oracular assurance, which proficients in either of these departments of inquiry will strive in vain to acquire. I need not say to you that, as students of the recondite sciences collectively styled medicine, such self confidence is unworthy of you. As Lord Bacon suggests, you can no more enter this earthly kingdom of knowledge, than the celestial kingdom of Heaven, without first becoming as little children. Your minds must be ready to receive truth as propounded. If you are deceived by your instructor, wittingly or unwittingly, the chances are thousand-fold that this will be less frequently repeated, than if you undertook your own guidance. It is a proverb among the lawyers, that he who undertakes to conduct his own case has a fool for a client, and the professional student who at the outset of his career determines to follow the uncertain lead of his own abortive notions will, sooner or later, find that he has undertaken a task which does more credit to his will than to his judgment or understanding.

Thus, upon the immediately practical branches which will here be taught, it must be insisted that they be fully received for your present guidance, whether in the practice of medicine, of surgery, or obstetrics, until after having acquired a competent acquaintance with all the necessary preliminary facts, you become qualified to rear the superstructure of your own opinions upon them. And in this connection the second proposition heretofore laid down becomes immediately important.

If your general education thus far has been properly conducted by your several preceptors, you have been taught how to use the mind in the acquisition of knowledge. Incident to the discipline required for this purpose, you have acquired some little store of knowledge. You have received many facts and principles likely to prove of direct service to you, but a large proportion of your studies have not had this object. Strength, and knowledge how to use it, have been the purpose.

If you have been conducted into the higher realms of mathematical study, it has been not that you should bring thence materials for every day use, but that your minds might come back firm, and exact and strong. If you have been led among the works of classic times, and devoted many toilsome hours to form an acquaintance with the languages in which they are written, it has not been that you may interlard your written or spoken words with choice phrases from comparatively unknown tougues, or that you might use them for every day's service, but that you might acquire keenness of perception, sharpness of discrimination, power of analysis, clearness, copiousness and accuracy of expression—faculties which may be cultivated by other means, and in which individuals may naturally differ, but which the experience of the world has shown to be peculiarly advantaged by this particular method. And so when you enter upon a course of study in the medical college, a very large proportionate object is, that you may be taught how best to employ your faculties in the future attainment of medical knowledge. We of the corps of instruction shall gladly point out what we believe the right and true path, accompany you a little way along its course, and then bid you God-speed. We shall do our best to instruct you in the facts of the science; we shall do our best to instruct you how to put them together and make use of them; how to think of them; but we forwarn you that then comes your own struggle, from which there can be no escape.

*Per aspera ad astra* is written all along the path of human endeavor. There is no royal road to knowledge, either medical or other. There is no cunning device of man, no adroit mechanism, no shrewd and shapely plan or scheme or programme which may be conceived, or written or printed, which will relieve you, or any other man or men, from the necessity of struggling and toiling and working, if you would ascend upward toward the temple of knowledge. In these latter times we hear much of adroit methods by which "knowledge is made easy." To hear some men, even of our profession, talk, and to read their writings, you would half expect that, by means of cunning methods of reform, the whole science of medicine could be wrapped up in a yellow-covered pamphlet, and, like "French without a master," be imparted in "six easy lessons."

On the other hand, there are those who would undertake to convince, that the advances in our profession have been such within the latter years, that it is a hopeless task for the student to undertake to acquire an acquaintance with them by any of the means which have hitherto received the sanction of the medical world. A new and royal road must be constructed—a kind of railroad to the hill-tops of knowledge—upon which the medical neophyte may take passage at a low rate of fare, and by the aid of numerous conductors, engineers, brakemen and switchmen, be conveyed to the *ne plus ultra* of medical wisdom —*cito, tuto et jucunde*.

With the ideas in the ascendant which we have heretofore laid down, you will at once perceive that with such fancies as these we have no sympathy—no affiliation.

How much that is indisputable and necessary to attainment, we have endeavored briefly to indicate. This is, always has been, and always will be true. There is no more of a burden laid upon the shoulders of this than of any preceding generation. Neither is there any less, nor will there be. Science has advanced with wondrous rapidity within the years that are past, but we have neither more nor less to acquire than our fathers had. It is a peculiarity of science, that while it is constantly accumulating to itself, it is also constantly separating from itself. The worthless, the cumbersome untruth is cast out as effete.

Take any branch of science you choose, and observe its method of advance, and see if this be not true in general and detail. I have sometimes thought truth might be compared to the statue within the as yet untouched block of marble—all that is wanting is for the master's hand and chisel to remove the rough and worthless material about it—to remove the surroundings which hide and smother it. And thus here in medicine, we glory in the fact that the advances made are not so much in the accumulation of rubbish which has to be removed and piled away by the pupil, but rather consists in actual removal of the rubbish, and more distinct approach to the truth in its simplicity.

And here I cannot forbear to suggest another idea which occurs to me, although it may appear to have a wider scope than the topic directly under consideration. It is this. Old and tried methods of acquiring knowledge which have stood the test of all varieties of innovation and attack, and which have, confessedly, produced such an intellectual and scientific harvest as the world has never before seen, ought not to be discarded or denounced, unless in favor of new methods of unquestioned and unquestionably great superiority. The danger is too vast to be incurred without positive promise. Confining this idea to medicine, and I must be allowed to observe that the present state of the science, and the brilliant catalogue of names of high worth, distinction and promise, which it presents to our view, whilst they may well cause our hearts to thrill with exultation at the success attained, inculcate, in the most impressive manner, an imperative caution lest we open the door to causeless innovation, and instead of "elevating the standard," we "hang a banner upon the outer wall," which shall speedily be prostrated amid utter confusion and turmoil.

I have stated that an acquaintance with the operations of other men's thoughts on this subject is important to the student. The advantage of this is twofold—it teaches what to do and what to avoid. Although the minds of men are not identical in action, they so far resemble each other that we may reason from one to the other with very great certainty. There is not an error afloat at the present time but what has its prototype upon the page of history. Perhaps we may say that there is not a truth now received, but what a more or less clear or dim apprehension of it has occurred to minds long since departed.

Many an inventor, ingenious, sagacious, skilful, has wasted his life in attempting combinations which a brief visit to the inventor's room at the Patent Office would have shown him to be fruitless, or, at the best, previously anticipated. And thus in the history of medicine we have a museum replete with instruction. Here you may derive hints which shall guide you to great discoveries—here you will find wrecks and beacon lights which shall preserve you, not only from attempting the impossible, but also from attempting the possible by impossible methods.

Here you will find thickly recurring evidences that that genius, so much coveted by men in general as enabling them to clear by single strides the long and tedious road of study, in far the greater proportion of cases has proved a bane to its possessor. The pages of that history will convince you, if nothing else will, that distinction, whether in the science or art of medicine, in the vast majority of cases, is due not to genius, not to especial advantages in the paraphernalia of education, but to a constant, assiduous devotion to the acquirement of knowledge and the cultivation of right modes of thought, so as to know the proper use to make of it when once acquired.

We invite your attention, Gentlemen, to difficult studies, vast in extent, varied in character, and yet, by the peculiar nature of the subject, acquaintance with each is indispensable to correct knowledge of either. For, let me forewarn you, that although for convenience, and of necessity, we assign particular branches to individual instructors, yet this division is almost wholly arbitrary—the parts are all of one body. Hence it is that instruction upon them is carried on, in point of time, as nearly concurrently as possible. Separation of these departments from each other, whether elementary or practical, is much like the Egyptian method, which appointed a medical custodian for each member of the body, and precluded each from meddling with the province of the other. Oftentimes the patient died, and all Egypt was startled to find that, as in case of railroad and steamboat accidents at the present time, the verdict of the doctors sitting each upon his particular organ, was, "None of these to blame." One prominent advantage of the usual lecture system adopted in this country is, that by this course we are enabled to keep the body of the science together, you are not obliged to gather up its mangled fragments, scattered along a couple or more of years.

Those of you who are attending the medical course for the first time will obtain a more general view, which will guide your studies to better advantage, which will suggest to you what points are especially noticeable and important, and when next you meet with the instructors of this, or some other college, you will be enabled to advance from generals to particulars, and with proper diligence will readily grasp and retain all or nearly all, that your teachers have opportunity to convey. And you need not fear to acquire everything which can be possibly imparted in the medical curriculum—you need not fear lest having overcome everything here, like Alexander, you must weep because you have no other worlds to subdue!

Above and beyond all this, you will have an open field for the exercise of every energy, of every faculty you may possess. The particular mode of imparting instruction usually adopted by the medical colleges of this country, and also throughout Europe, although it has been sharply inveighed against, and attacked from various quarters, we must still believe to possess peculiar and marked advantages.

Oral instruction, as communicated in the lecture room, calls into active exercise the faculties of attention and perception—faculties of the highest importance to the future practitioner as well as the student. A few weeks' experience will prove to you that the discipline thus afforded is of the most valuable character. At the outset, perhaps, you may feel discouraged, possibly disheartened, at the idea of attempting to follow along with the rapid procession of truths which will, hour after hour, and day after day, be moving on and on. But let me reassure you, and call for the testimony of those who have gone before you in the same course. The task will daily become easier, and before the lapse of one-third or one-half the session, you will find it a matter of little or no difficulty.

There is an intimate relation which gradually becomes closer and closer between the lecturer and his class—scarcely to be explained by words or philosophy, but every teacher, every student readily appreciates it. The instructor in communicating knows whether he is understood; he can read his own thought mirrored in the eyes, photographed upon the features of his auditors. If that mirror fails to reflect the idea, if that moving feature fails to give back the form thrown upon it, the object will again and again be held up till the mental light has fixed it forever. Here books fail—here the speaker triumphs. And the triumph is not his alone, for the new idea gained is the student's victory also—it is one step further toward the ultimate goal of knowledge—it is another assertion of the supremacy of man over ignorance and folly and delusion. Count me the value of a single truth, and I will multiply it for you into the sum of all values which separate the civilized and enlightened Anglo-Saxon from the Hottentot of the Cape!

And permit me to say, Gentlemen, in this connection, that even the number of lectures to which you will daily listen are so apportioned as to meet the best uses of the mind in discipline. Six lectures a day, of an hour each, are no more than a healthy and moderately well disciplined mind requires to bring out its true power. Science brooks no indolence in its votaries, and slow movements are abhorrent to fleet stepping truths.

It is not the mere acquirement of knowledge which is wanted—it is something more—it is culture and power. A Hercules is not brought to his full stature by careful feeding, nursing and seclusion from effort. Athletæ are not so produced. Muscles must be exercised daily to their utmost in order to full development, and so must brains. The oak may thrive in a hot house, but it will lose many of its most admirable characteristics. If it would bid defiance to the storm, it must from its infancy be *subjected to the storm,* so that its roots shall sink deeper, and take firmer hold upon the soil and its rocky substratum.

And so of the human mind, if you would have it developed into all the majestic proportions of which it is capable, you must, from the earliest dawn of its development to the final hour, tax all its energies, all its capacities.

Pressure is what is needed—collision with all the wants, anxieties, ambitions and cares of life. Troublous times and imminent crises in the affairs of nations produce harvests of great men—"piping times of peace," luxurious ease and philosophic retirement, straggling weeds of littleness only. None of you rightly know what you can do until stern necessity, or fierce ambition, compels to the effort; and you are angels, and not men, if you do what you might without.

My friends, have you ever thought what this human mind of ours is, or even what it is like? Have you measured its capacities, and marked the limit of its growth? Have you ever thought that from the cradle to the grave it grows unceasingly, and commands to itself the servitude of all of existence, whether seen or unseen, from the next material object to the coming light of the farthest star?

The scriptural injunction is not only to do certain things, but "whatsoever thy hand findeth to do, that *do with thy might.*" Put all your power, not only of muscle, but of heart and brain, into the work.

We expect, Gentlemen, to fill up all your time with work. We do not call upon you to idle away an hour; we propose to ascertain what manner of men you are. We want for this profession of ours recruits that not merely understand tactics, but are capable of drill and service, with iron muscles and active brains. Let the sick men and the children, who cannot keep up with the grand army, fall back into the rear with the women, the sutlers and the baggage.

And the history of the profession bears us out in the doctrines we advance. Who are the stars of the profession in this country and throughout the world at this hour? Those who feebly complain that too much labor was imposed upon them during their pupilage? Not one—not one! But those who wrought with a will at the work before them, and rejoiced at the very burdens imposed. This is the only way to bring out the true man. Why, you can only get at the truly gigantic force of steam when you bear it down and put your weights upon it, cage it up in iron walls and put fire under it—then you will find the secret of its power, and, wo to the unlucky wight who does not "look out for that engine when its bell rings!"

Very well intentioned gentlemen, easy going gentlemen with pens in their hands and crotchets in their heads, in very good and grammatical English, and in very bad and ungrammatical attempts at English, have duly expressed their profound concern at the dubious character and prospects of the rising medical generation. The nervous system of this new generation is too delicate to be subjected to the stern labors and rigorous processes which their fathers endured. Indeed, it has become quite a fashionable mode of securing reputation as a sage, to diagnose in the bodies of juvenile candidates for the profession weaknesses which must preclude them from undergoing that discipline which made their fathers staunch and strong. We do not so believe—for the credit of manhood we will not so act.

*Haud inexpertus loquor*—from my own personal observation I am ready to give evidence, that the number of lectures *per diem* here given is not only not too much for the student, but, taking the average, it is much better for him than any less number. Let the profession give testimony. One other point, in this connection, by way of explanation and encouragement. The different branches of medical study are so intimately related to each other, that they mutually facilitate clear understanding and recollection. Thus, of the large number of facts which will day by day be brought to your notice, a very large proportion will actually serve as helps to the memory in retaining the others. This is peculiarly the case with observations in clinical practice in the various hospitals to which you will here have access, and in the variety of cases which will from time to time be brought before you in this building. To use a homely figure—each case will furnish you a string upon which you can collect in one view many important facts, anatomical, physiological, pathological, therapeutic or surgical, as the case may be. The clinic will thus afford you a faithful and reliable mnemonic chart, infinitely preferable to the absurd and unphilosophical schemes which mere memorizers are so constantly inventing. For, after all, the clinic is the soul of the body of instruction—our art and science culminate in the cure of disease.

You are to look upon hospital and other observation, therefore, not as adding to your labors, but as facilitating them. Few persons seem to understand the true import of the faculty of memory. We hear many complaining of the want of this power, and we are often ready to complain of its exuberant manifestation. May I venture to impart a secret? That which is thoroughly mastered and understood cannot be forgotten, and on the other hand, that which you can neither master nor understand, had best be forgotten as speedily as your mental constitution will permit. A muddy, half conception of a truth is worse than none at all, and it is notorious the world over, in all points of belief, that no doctrines are so strenuously adhered to, however absurd in themselves, as those which have some shadow of truth in their foundation. The meagre half-truth, after a very aboriginal fashion, is made the vehicle or trumpet of a horde of plethoric falsehoods.

Need it be said that a clear apprehension of the facts and principles which you will here be instructed in, will forever preserve you against falling away into the belief or practice of any of the multitudinous varieties of medical delusion or imposture with which the world is full?

I congratulate you, Gentlemen, that you have commenced the study of a profession wherein is permitted the most complete independence of thought. We have here no edicts which control unalterably, save those which science itself imposes. You are free to think and judge in medicine, as in any of the natural sciences—no more, no less.

And yet we are proud to assure you that ours is a conservative profession. The new fact, or new principle, which would find place in our creed, must come thoroughly demonstrated and proven by all the tests which modern science affords—no more, no less.

Whilst we have signed a declaration of independence against ancient dogmas, we nevertheless refuse to be dragged as willing dupes at the chariot wheels of every loud-voiced imposture, although it labels itself upon its brazen forehead—*Progress*. Asserting freedom, we avoid that anarchy of opinions wherein we should neither be governed by others nor by ourselves.

Of this anarchy of minds there are, alas, multitudinous examples in these our times, beset with changes which are progress, and changes which are not progress, but a miserable retrograde towards barbarism.

The street corners are vocal with noisy declamation of pseudo-reformers; the books and newspapers overflow with the inky diatribes of those who would re-create the world after models which it would be no sin to worship, being unlike anything in heaven, on earth, or in the waters under the earth. It is an unfortunate fact, that many truths in government, religion, science and art have only been received after great opposition, by the world, and hence at the present time, there is not an impudent quack or arrant knave, tampering with either of these, but congratulates himself on the kicks and cuffs he receives from all sober-minded, thinking men, because he can use them as arguments that he too is a great reformer, very badly treated, and therefore in the right!

Poor old Galileo was sent to prison because he saw fit to make use of the earth's revolution upon its axis, as an argument against certain doctrines of the Church; and now there is not a pestilent disturber of the public peace, there is not an absurd fanatic, there is not a meddlesome demagogue, there is not a noisy mountebank, but when derided for his folly, dashes his heels against his ricketty wheelbarrow of notions, and *a la* Galileo, exclaims—"It does move, anyhow!"

Something more than opposition is required as a test, whether of truth or falsehood, as many in these days are beginning to find to their cost.

You may be independent men, but to be truly so, you must first be studious and thinking men. The mandate has come down from the earliest dawn of time, by the sweat of man's brow shall he live—live corporeally, live intellectually, live spiritually, and wo to him who disobeys.

Nor is there any real hardship in this, as the indolent and slothful imagine. Obedience to this law of our being converts the primal curse into a perennial blessing. The sluggard is but one remove from the vegetable—the earnestly industrious man advances day by day with ceaseless progression towards the attributes of the Divine.

It is work, effort and toil which have mapped the continents with cities and towns and hamlets; which have replaced the wilds with cultivated farms, and the void deserts with all the arts and busy hum of civilized life; which have "spanned the rivers with great bridges unknown to our fathers;" have traversed the seas with sailless ships; have covered the earth with railways and telegraphs; have carried the sciences to a meridian splendor, and have made man truly—"In action, how like an angel! in apprehension, how like a god!"

It is a proud reflection, Gentlemen, that our profession has always occupied a distinguished place in the van of the world's onward march. All along the glorious catalogue of those whom the grateful ages "will not willingly let die," the names of medical men glitter as "bright particular stars," and, to-day, no higher homage of admiration and respect is paid to any other than those which our profession claims as its chosen ones.

Disappointed aspirants for fame on the basis of slight attainments and pauper intellects—misanthropes overborne by dyspepsia and hypochondria—enthusiastic dreamers of Utopia—these and the like may ventilate their ululations over the degradation of the profession, but to these we have no response save an indignant denial of both their premises and their conclusions.

We welcome you, Gentlemen, to no degraded position, but to one which of itself merits and receives honor, and to which we trust you will hereafter bring additional reasons for popular confidence and respect.

Beyond this we may say, that in the professional ranks you will be allotted a place entirely commensurate with the intellect, culture and acquirement you bring with you. On the progress you here make in medical science will depend much of your ultimate success. Endeavor to lay the foundation broad and deep, that you may build vast and high. Aim at a clear understanding of the subject, rather than mere memorizing of details. Seek strength and solidity rather than words and phrases. The time will come when the parrot tongue will not answer—*brains* must have to do with the work. The alphabet and the dictionary must be passed by for the book which may be known, and read of all men.

To whatsoever we, your temporary instructors, can do to advance you towards eventual and deserved professional success, we bid you earnest, heartfelt—Welcome!

